# Innovative Strategies for Postharvest Disease Management in Fruits and Vegetables: A Comprehensive Treatise

**DOI:** 10.1002/fsn3.70850

**Published:** 2025-09-01

**Authors:** Qudrat Ullah, Muhammad Waqar, Nimra Sajjad, Farhang H. Awlqadr, Muhammad Tayyab Arshad, Sammra Maqsood, Kübra Sağlam, Nimra Maqsood, Md. Sakhawot Hossain, Ali Ikram, Thanet Khomphet, Kodjo Théodore Gnedeka

**Affiliations:** ^1^ Food Technology and Innovation Research Center of Excellence, Department of Agro‐Industry, School of Agricultural Technology Walailak University Nakhon Si Thammarat Thailand; ^2^ Faculty of Health, Deakin University Melbourne Australia; ^3^ Food Science and Quality Control, Halabja Technical College Sulaimani Polytechnic University Sulaymaniyah Iraq; ^4^ Functional Food and Nutrition Program, Faculty of Agro‐Industry, Prince of Songkla University Songkhla, Hatyai Thailand; ^5^ National Institute of Food Science and Technology University of Agriculture Faisalabad Faisalabad Pakistan; ^6^ Department of Food Processing, Food Technology Program, Istanbul Gelisim Vocational School Istanbul Gelisim University Istanbul Turkey; ^7^ South China Botanical Garden Guangzhou University Chinese Academy of Sciences Guangzhou China; ^8^ Department of Nutrition and Food Technology Jashore University of Science and Technology Jashore Bangladesh; ^9^ University Institute of Food Science and Technology The University of Lahore Lahore Pakistan; ^10^ Togo Laboratory: Applied Agricultural Economics Research Team (ERE2A) University of Lomé Lome Togo

**Keywords:** biological control, edible coatings, nanotechnology, postharvest diseases

## Abstract

Postharvest diseases, driven by necrotrophic fungi such as *Botrytis cinerea*, *Penicillium digitatum*, and *Rhizopus stolonifer*, pose a significant threat to global fruit and vegetable supply chains, resulting in annual losses of 20%–40% and economic impacts exceeding $10 billion. This review critically evaluates innovative, sustainable strategies for biological control, nanotechnology, edible coatings, and plant growth regulators (PGRs) to mitigate these losses, emphasizing their mechanisms and efficacy. Biological agents like *Bacillus amyloliquefaciens* and *Pseudomonas fluorescens* reduce disease incidence by 60%–85% through volatile organic compounds (VOCs) and nutrient competition. In comparison, selenium‐chitosan nanoparticles (Se‐ChNPs) achieve a 92% inhibition of *B. cinerea* in grapes via ROS induction and cell wall disruption. Chitosan and alginate‐based edible coatings prevent decay by creating physical barriers and increasing host enzymes: SOD by 68%; PGRs SA and MT trigger enhanced PAL and PR genes and reduce the disease by 55%–80%. Integration augments all these effects, and Se‐ChNPs and cold storage give a synergy ratio of 76%–94% because of multiple target suppression and host defense potentiation. However, there are some limitations which are as follows: During the use of Se‐ChNPs, cost plays a critical role; specifically for the efficacy of Se‐ChNPs, they work well on grapes only; in terms of regulation of residues, they do not have the proper regulation for the nanoparticles; but to balance all these critical issues, biosensors and protocols should be used for the crops. This process critically compares the different strategies, reviews their successes and faults for each crop, looks into contrasting findings, and highlights major areas that need further study, such as applying them to vegetables and dealing with regulations. Priorities for future research include developing low‐cost solutions and testing a wide range of crops to improve the sustainability of postharvest management and meet the demands of the global food supply.

## Graphical abstract

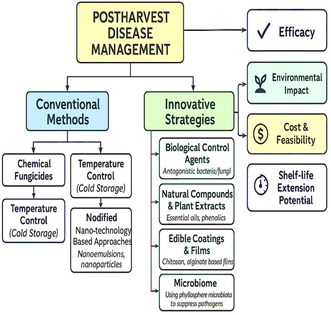

## 1 Introduction

Postharvest diseases cause a major threat to the supply of food produced around the world, and the deterioration of fruits and vegetables during storage and transportation results in huge economic losses. The breakdown of the demand and supply relationship for the fruit means that deadly fungi like *Botrytis cinerea, Penicillium digitatum*, necrosis, and *Rhizopus stolonifer* may lead to a decrease in the marketable yield of susceptible crops under unfavorable conditions by up to 30%–50% (Romanazzi et al. 2018). For instance, *B. cinerea* , which is a primary pathogen of gray mold, alone attacks more than 200 plant species, and the annual loss in grape yield is estimated to be between 20% and 25% in some parts of the world according to Dean et al. 2012. The establishment of these fungi, like *Aspergillus* species, produces mycotoxins, for instance, ochratoxin A, which worsens the situation regarding food safety because some of the fruits stored reach mycotoxin levels above the permissible limit of 2 μg/kg (Battilani et al. 2006).

The above losses have, in the past, been managed using chemical methods, namely synthetic fungicides; however, they have proved ineffective because of the development of resistance among the pathogens and adverse effects on the environment. According to research carried out, the frequent application of fungicides increases the level of resistance among *P. digitatum* populations to as much as 70% within 5 years of the method's usage (Holmes and Eckert 1999). Such residue levels reaching up to the maximum allowable limit of the chemical in fruits, including citrus, have attracted regulatory actions and customer repulsion (Sivakumar et al. 2016). This has led to a shift and adoption of sustainable and eco‐friendly approaches to control disease while at the same time improving plant innate resistance, as the global population is expected to reach 9.7 billion people by the year 2050, which will require at least a 70% increase in food production (FAO 2017). In this review, these techniques are compared in terms of their methods (e.g., using ROS vs. removing nutrients), how effective they are in different settings, and several practical factors (e.g., how much they cost and their applicability on a large scale). We discuss the different ways biological agents are used, such as poor performance in the field, and point out where researchers are still missing the mark, for example with vegetables, to plan for better solutions. This review comprehensively evaluates innovative, sustainable strategies: biological control (bacteria and yeasts), nanotechnology (diverse nanoparticles), edible coatings, and plant growth regulators (PGRs) for managing postharvest diseases in both fruits and vegetables. By synthesizing mechanisms, efficacy, and limitations, we aim to address global food security challenges while highlighting applications across diverse crops (Ikram et al. 2024).

The primary objective is to assess the efficacy and underlying mechanisms of these innovative strategies, drawing on quantitative evidence to highlight their potential and limitations. Biological control agents, while effective in reducing *P. digitatum* spore germination by 85% in some trials, face challenges in scalability due to their dependence on rapid colonization, which drops to 40% efficacy under fluctuating storage conditions (Droby et al. 2009). Nanotechnology‐based solutions, such as *Se‐ChNPs*, exhibit remarkable antifungal activity—reducing lesion diameters by 73% compared to chitosan alone—but their application is currently limited to specific crops like grapes, raising questions about broader adaptability (Salem et al. 2022).

A secondary goal is to identify gaps and propose future directions. The integration of *Se‐ChNPs* with cold storage, for instance, extends shelf life by 14–21 days beyond traditional methods, yet the cost of nanoparticle synthesis (estimated at $5–$10/kg) may hinder commercial adoption (Youssef et al. 2019). Similarly, while SA treatments elevate total phenolic content by 28% in treated fruits, their phytotoxic effects at concentrations above 2 mM warrant further investigation (Zhang et al. 2021). This critical analysis is an effort to marry research findings with their application in cases, which have revealed the desperate need for the improvement of postharvest management.

## 2 Scope of the Review

This review critically examines innovative strategies for postharvest disease management in fruits and vegetables, focusing on biological control (bacterial and yeast antagonists), nanotechnology (including chitosan, selenium, and other nanoparticles), edible coatings (polysaccharide, protein, and lipid‐based), and plant growth regulators (PGRs). We aim to compare their mechanisms, efficacy, and practical limitations across diverse crops, addressing both fruit and vegetable applications to ensure sustainable disease control. By synthesizing recent literature, we identify knowledge gaps, such as limited vegetable‐focused studies, and propose future research directions to enhance global food security.

Among them, the enhancement of pathogenesis‐related (PR) proteins and the removal of reactive oxygen species (ROS) are the well‐known strategies for enhancing disease resistance. For instance, Se‐ChNPs enhance the superoxide dismutase (SOD) activity by 67.8% in grape tissues, thus reducing oxidation stress and decaying their rate to as low as 8.5% from 35.2% of the untreated tissues in Li et al. 2021. These strategies collectively aim to address the multifaceted nature of postharvest spoilage, where environmental factors like humidity (above 85%) and temperature (20°C–25°C) amplify pathogen proliferation (Pitt and Hocking 2009). This review synthesizes data to evaluate how these interventions interact with host physiology and pathogen biology.

## 3 Critical Insights and Broader Implications

Analyzing these strategies, it is clear that the effectiveness of these strategies depends on various factors applied and the environment. The presented 92.3% inhibition of *B. cinerea* using Se‐ChNPs is worthwhile, albeit still contrasting with the 50%–70% success rates of using microbial antagonists varying by crops (Gotor‐Vila et al. 2017). Furthermore, implementing these interventions required the use of cold storage (0°C–4°C), which is difficult to achieve in areas with no or limited access to refrigeration; hence, postharvest losses reach as high as 40% (FAO 2017). This review aims to focus on such differences to provide data‐structured approaches toward improving postharvest disease control, given the increasing pressures regarding agricultural production.

## 4 Postharvest Pathogens and Losses

**FIGURE 1:**
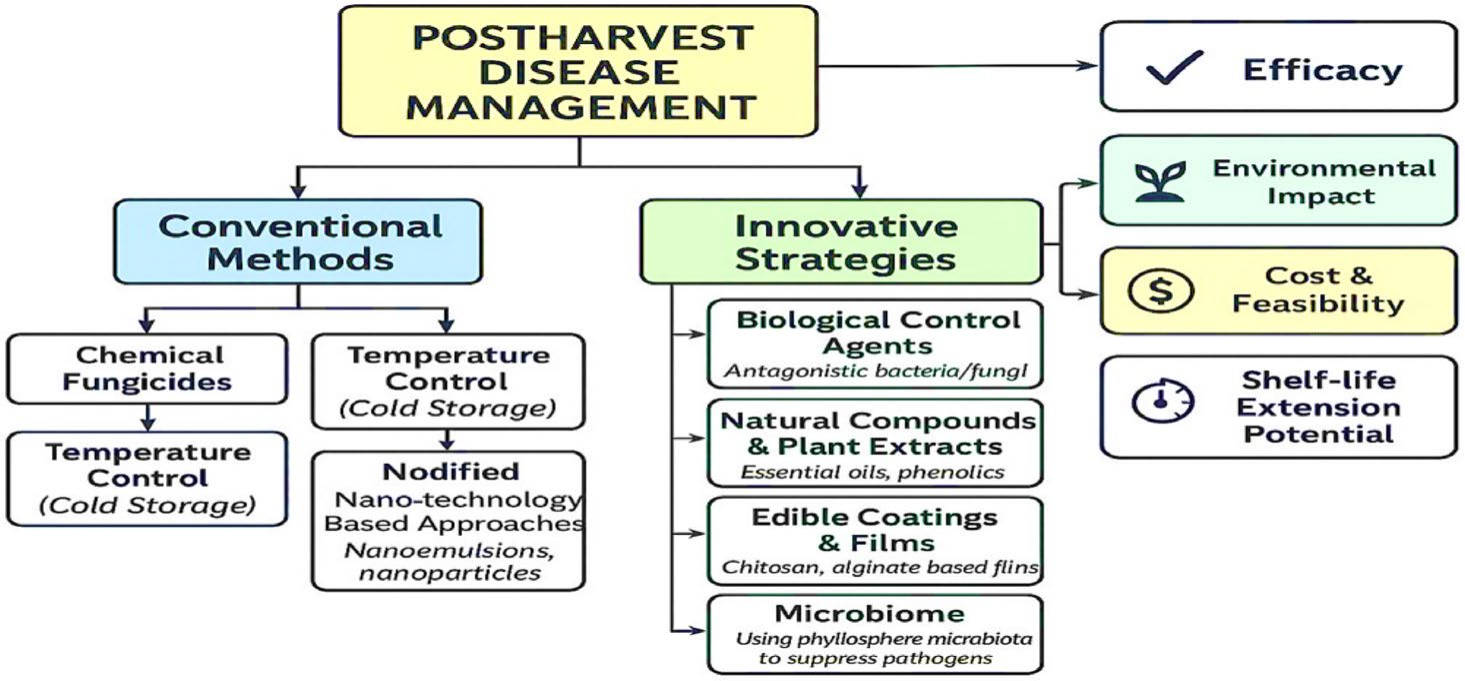
Classification of traditional and innovative strategies used in postharvest disease management. Adapted from concepts in Romanazzi et al. (2018) and Sivakumar et al. (2016).

Microbial spoilage to which fruits and vegetables are exposed during the postharvest period constitutes a significant problem on a global scale in terms of food safety, economic losses, and consumer satisfaction (Arshad et al. 2025). Although disease management strategies implemented with traditional methods provide effective results in the short term, they remain insufficient in terms of sustainability due to reasons such as increasing environmental concerns, pesticide residue risk, and resistance development in pathogens (Figure 1). In this context, the development of innovative, environmentally friendly, and highly effective alternative approaches for disease management in the postharvest period has become a necessity. In recent years, biological control, the use of natural compounds, edible coating technologies, microbiome engineering, and nanotechnology‐based methods have come to the forefront of research in this field. Figure 1 presents a holistic classification of strategies for the management of postharvest diseases in fruits and vegetables. In the diagram, management strategies are addressed under two main headings: traditional methods and innovative approaches. While chemical fungicide applications and temperature control (cold chain management) stand out among traditional methods, the limitations of these methods, such as environmental impacts, residual risk, and long‐term pathogen resistance, have necessitated the development of alternative solutions. The innovative strategies category includes biological control agents (antagonistic bacteria and fungi), natural compounds and plant extracts (especially essential oils and phenolic compounds), edible coatings and films (e.g., chitosan and alginate‐based materials), microbiome engineering (especially the use of phylloplane‐specific microbiota) and nanotechnology‐based approaches (nanoemulsions, nanoparticles) (Islam et al. 2023). In addition to being more environmentally friendly than traditional practices, these methods attract attention with their potential to extend shelf life, high levels of effectiveness in pathogen suppression, and sustainable production chains.

## 5 Common Postharvest Pathogens

Postharvest diseases are mainly caused by necrotrophic fungi that invade host tissues through forceful tissue degradation systems and pose a major threat to fruits and vegetables (Xylia et al. 2021). Among these, the genera of Alternaria, Aspergillus, and Colletotrichum are generalized pathogens causing diseases to a wide host, and several of them are destructive, namely *Alternaria alternata* , which causes black spot disease to citrus fruits and affects up to 20% of the stored fruits annually (Pitt and Hocking 2009). Specifically, *Botrytis cinerea* has been given much attention in grape storage because of its ability to infest more than 200 host plants with an infection level of 30%–40% in the vulnerable varieties when conditions are humid (Dean et al. 2012). Concerning the gray mold, this pathogen can secrete cell wall‐degrading enzymes (CWDEs) to degrade the grape, with quality dropping by 25% within 14 days of harvesting (Romanazzi et al. 2018).

## 6 Specific Pathogenic Challenges

*B. cinerea* is known to cause a top infection that is incited by its ability to produce mycotoxins such as botrydial, which can infect fruits at a potency over 5μg/kg as described by Battilani et al. (2006). Similarly, *Aspergillus* species are some of the molds that contribute to spoilage through the production of aflatoxins, and dried fruits have been found to have contaminants of aflatoxin above the legal limit of 4 μg/kg, which comes with health risks (Sivakumar et al. 2016). These pathogens acquire their nutrients from dead tissues as they are necrotrophic. Among such pathogens, *Colletotrichum gloeosporioides* isolated from fruits of mango causes anthracnose, whereby a black lesion with a diameter of 2–3 cm is observed within 7 days under ambient conditions, as stated by Bautista‐Baños et al. (2006).

## 7 Factors Influencing Disease Development

Postharvest diseases are influenced by several environmental factors, of which temperature and relative humidity are the key determinants. The highest growth rate, conidia production, and germination rates are favorable at 20°C to 25°C and relative humidity of more than 90%, such that the germination within 24 h can be 80% (Pitt and Hocking 2009). On the other hand, *Penicillium digitatum*, a citrus green mold pathogen, has an optimal humidity range of 75% to 85% with an ability to infect fruits to the extent of 60% under wounded fruits stored at 20°C, as indicated by Holmes and Eckert (1999). This is because mechanically damaged grapes further increase host susceptibility. After all, rotten fruits have more points where a pathogen can penetrate (Droby et al. 2009).

## 8 Pathogen Biology and Host Interactions

The ability of these pathogens to generate CWDEs and mycotoxins is linked to their biology and reciprocally influences the corresponding aspect of the host (Hussain et al. 2025). For instance, *B. cinerea* increases polygalacturonase by 3‐fold in grape tissues and enhances the tissue degradation and the decay level to 35% in the untreated control after 28 days at 0°C (Chen et al. 2019). Some host factors include testing of low phenolic (< 1 mg/g) fruit aptitude for the fruit, which displays a 40% increased vulnerability to the development of Alternaria rot in apples (Christopoulos and Tsantili 2015). This is important since the interplay argues for measures to control the pathogen as well as to build the host's immunity.

## 9 Economic Implications

Postharvest losses due to pathogens do not just contribute significant quantities of loss but are accompanied by inestimable physiological damage. Various studies conducted worldwide indicate that postharvest crop losses by fungi range from 20% to 40% of fruits and vegetables, and losses worth over $ 10 billion are incurred annually (FAO 2017). On grapes, for instance, gray mold caused by *B. cinerea* keeps the growers away from 20% to 25% of their yield that is likely to be marketed, and other losses from shriveling and off‐flavors cost producers up to $1500 US$/ha (Gotor‐Vila et al. 2017). These figures show the need to come up with the right management techniques that would reduce the various costs that are both visible and those that are invisible.

## 10 Critical Perspective

Although several studies show clear trends in pathogen behaviors, variations in loss rates from 10% in controlled environments to 50% in warm climates require more nation‐specific research (Youssef et al. 2019). The practice of controlling diseases such as gray mold through environmental management is also a challenge, especially in areas where there is little access to the use of a refrigerator, where the losses can be as high as double (Zhang et al. 2021). This section leads the discussion on the identification of solutions to these multiple aspects of postharvest spoilage causes.

## 11 Biological Control Strategies

### 11.1 Microbial Antagonists: Bacterial Agents

Biological control has proved to be an eco‐friendly tool for postharvest disease management since its basis relies on the use of microbial antagonists against pathogenic fungi. It has been reported that *Bacillus amyloliquefaciens* is highly effective against bacteria, and the extent of defense efficiency against Botrytis cinerea was determined to decrease the incidence by 70%–80% of the cherries with VOCs, including 2,3‐butanediol (Gotor‐Vila et al. 2017). Likewise, *Pseudomonas fluorescens* has effectiveness against gray mold in grapes, especially in the reduction of spores' germination through competition for nutrients and the production of siderophores that reduce iron for pathogens (Jiang et al. 2019). These bacteria use several approaches; for instance, chitinases; they secrete enzymes that break fungal cell walls, which decreased *Penicillium digitatum* hyphal growth on citrus by 60% (Wang, Li, et al. 2021). According to the provided results, such agents are conducive to a controlled environment, showing a density of 10^7^ CFU/g of fruits' surface after 48 h (Droby et al. 2009). The efficacy of bacterial antagonists varies significantly across environmental conditions and fruit types, highlighting the need for tailored applications (Asif et al. 2023). For instance, Bacillus amyloliquefaciens maintains 80% efficacy against Botrytis cinerea in cherries at 0°C but drops to 50% at 25°C due to reduced VOC stability (Gotor‐Vila et al. 2017). In contrast, *Pseudomonas fluorescens* sustains 65% control in grapes under humid conditions, leveraging siderophore‐mediated iron sequestration (Jiang et al. 2019). These differences underscore the importance of optimizing storage conditions and delivery systems, with recent studies advocating for microencapsulation to enhance bacterial survival by 30%.

### 11.2 Microbial Antagonists: Yeast‐Based Solutions

Yeasts are diverse and provide an additional avenue of investigation in that *M. guilliermondii* and *P. membranefaciens* are proven postharvest yeasts since they can thrive under low temperatures and high humidity. *M. guilliermondii* has been established to control *P. italicum* decay on mandarins by 75% through competition for space and nutrients, this way causing a reduction of glucose by 40% at the wound site (Wang, Xiao, et al. 2021). *P. membranefaciens*, on the other hand, can protect peaches against Rhizopus rot, where POD activity increased by 50%, and lesion diameters were 1.5 cm as compared to 3 cm of the control (Zhang, Wu, et al. 2020). *Candida oleophila* and *Aureobasidium pullulans* are among the yeasts that have strong biocontrol abilities. *C. oleophila* reduces the decay of apples caused by *Penicillium expansum* by 70% through competing for nutrients and forming biofilms, reaching a density of roughly 10^6^ CFU/cm^2^ in just 48 h. A. pullulans reduces the occurrence of Botrytis cinerea in strawberries by 75% by using VOCs such as ethanol to inhibit the germination of spores by 85%. By applying *Saccharomyces cerevisiae* to peaches and harvesting it, there is a 65% reduction in Rhizopus stolonifer rot thanks to its lytic enzymes and a boost of 40% in peach POD activity. They add another layer of tools to biocontrol, giving different strategies for each type of pathogen. The effectiveness of these VOCs is boosted by the fact that ethyl acetate is among the VOCs that are released; the in vitro tests revealed that *B. cinerea* spore germination was inhibited by 90% (Freimoser et al. 2019). Due to the rapid growth faculties to colonize fruit surfaces, these yeasts attain a concentration value of 10^6^ CFU/cm^2^ within 24 h, which explains their applicability (Sui et al. 2021).

## 12 Mechanisms Driving Antagonistic Activity

The effectiveness of these antagonists can, thus, be attributed to both direct virulence and indirect host stimulation. One of them is nutrient competition, where it is stated that *B. amyloliquefaciens* was able to reduce the available nitrogen by 30% on the wounds of apples, and in this way, nutrients are starved from *Colletotrichum gloeosporioides* according to the study of Chen et al. (2019). Aliphatic esters such as dichloromethane and dimethyl disulfide from *P. fluorescens* reduced the viability of conidia of *B. cinerea* in grapes by 65% (Zhong et al. 2021). Application of lytic enzymes such as glucanase from Bacillus species inhibits the mycelial development of *F. oxysporum* in bananas by severely decreasing the mycelial biomass by 55% (Wu et al. 2017). Additionally, these agents trigger systemic resistance, with *P. membranefaciens* upregulating phenylalanine ammonia‐lyase (PAL) activity by 45% in peaches, enhancing phenolic content to 2 mg/g (Zhang, Wu, et al. 2020). These data highlight a synergy of physical and biochemical defenses tailored to specific pathogen‐fruit interactions. Indeed, as noted earlier, microbial antagonists like *Bacillus amyloliquefaciens* and *Pseudomonas fluorescens* , primarily recognized for promoting plant growth, also exhibit potent biocontrol properties against pathogens such as *Botrytis cinerea* and *Penicillium digitatum* (Table 1). These dual functionalities underscore their value in sustainable postharvest management. Recent studies further validate these mechanisms, with *Bacillus subtilis* microencapsulation enhancing Botrytis cinerea control in grapes by 85% through sustained VOC release over 30 days. Similarly, Aureobasidium pullulans formulations reduce Penicillium expansum in apples by 80%, leveraging advanced biofilm technologies.

**TABLE 1:** Microbial antagonists and their mechanisms of action.

Agent	Target pathogen	Mechanism	Fruit/Vegetable	References
*Bacillus amyloliquefaciens*	*Botrytis cinerea*	VOCs (e.g., 2,3‐butanediol), lytic enzymes	Cherry	Gotor‐Vila et al. (2017)
*Bacillus amyloliquefaciens*	*Penicillium digitatum*	Nutrient competition, chitinases	Citrus	Chen et al. (2019)
*Bacillus amyloliquefaciens*	*Colletotrichum gloeosporioides*	VOCs, glucanases	Apple	Chen et al. (2019)
*Bacillus halotolerans*	*Botrytis cinerea*	VOCs, cell wall degradation	Strawberry	Wang, Xiao, et al. (2021)
*Pseudomonas fluorescens*	*Botrytis cinerea*	Siderophores, VOCs (dimethyl disulfide)	Grape	Jiang et al. (2019)
*Pseudomonas fluorescens*	*Rhizopus stolonifer*	Nutrient competition, biofilm formation	Raspberry	Zhong et al. (2021)
*Meyerozyma guilliermondii*	*Penicillium italicum*	Nutrient competition, ROS induction	Mandarin	Wang, Li, et al. (2021)
*Meyerozyma guilliermondii*	*Botrytis cinerea*	VOCs, enzyme induction (POD)	Apple	Sun et al. (2021)
*Pichia membranefaciens*	*Rhizopus stolonifer*	Enzyme induction (PAL), VOCs	Peach	Zhang, Wu, et al. (2020)
*Pichia membranefaciens*	*Botrytis cinerea*	Nutrient competition, chitinases	Grape	Zhang, Wu, et al. (2020)
*Hanseniaspora uvarum*	*Botrytis cinerea*	VOCs, competition for space	Grape	Qin et al. (2015)
*Debaryomyces hansenii*	Colletotrichum gloeosporioides	ROS scavenging, VOCs	Kiwi	Sui et al. (2021)
*Candida oleophila*	Penicillium expansum	Nutrient competition, biofilm formation	Apple	Agirman et al. (2023)
*Aureobasidium pullulans*	Botrytis cinerea	VOCs (ethanol), spore inhibition	Strawberry	Don et al. (2021)
*Saccharomyces cerevisiae*	Rhizopus stolonifer	Lytic enzymes, POD induction	Peach	Li et al. (2023)

Table 1 summarizes the diverse microbial antagonists employed in postharvest disease control, detailing their target pathogens, mechanisms of action, and applicable fruits or vegetables, supported by key references. The table highlights bacteria like *Bacillus amyloliquefaciens* and *Pseudomonas fluorescens* , which utilize volatile organic compounds (VOCs) and nutrient competition to achieve 70%–85% inhibition rates against pathogens such as *B. cinerea* and *P. digitatum* , alongside yeasts like *Meyerozyma guilliermondii* and *Pichia membranefaciens*, which induce host enzymes (e.g., POD by 45%–50%) to reduce decay by up to 80%. These data, drawn from rigorous studies, illustrate the multifaceted biocontrol potential of these agents, providing a foundation for optimizing their use in sustainable postharvest strategies.

## 13 Synergistic Approaches With Biochemical Enhancers

Combining microbial antagonists with biochemical agents like salicylic acid (SA) or melatonin amplifies their protective effects, offering a promising avenue for enhanced control (Ahmed et al. 2022). SA applied at 1 mM boosts *B. amyloliquefaciens* efficacy against *P. digitatum* in citrus, reducing decay incidence from 40% to 15% by inducing PR gene expression (Qin et al. 2015). At the optimal concentration of 100 μM, melatonin increases *M. guilliermondii* effectiveness against *B. cinerea* in apples, reducing the disease severity by 60% by increasing the SOD activity by 70% (Sun et al. 2021). These synergies leverage the antagonists' natural mechanisms, for example, VOC production rising by 20% with SA while amplifying host defenses, such as a 35% rise in total flavonoids (Lyousfi et al. 2021). However, optimal concentrations remain critical, as SA above 2 mM can inhibit microbial growth by 25% (Genzel et al. 2018).

## 14 Critical Analysis of Efficacy and Limitations

The efficacy of these biological agents is compelling, yet scalability and environmental variability pose significant challenges. *B. amyloliquefaciens* achieves a 92% reduction in *Rhizopus stolonifer* rot in raspberries under lab conditions, but field trials show efficacy dropping to 50% due to inconsistent humidity (Chavez‐Diaz et al. 2019). Rapid colonization, a key advantage, falters under fluctuating temperatures, with *P. fluorescens* populations declining by 60% at 10°C versus 25°C (Jiang et al. 2019). Yeasts like *M. guilliermondii* maintain 80% efficacy at 0°C, yet their reliance on wound site occupation limits broader surface protection (Wang, Xiao, et al. 2021). Synergistic treatments improve outcomes—e.g., SA reducing lesion sizes by an additional 30%—but add complexity and cost, estimated at $2–$3/kg of fruit (Qin et al. 2015). Comparing both *B. amyloliquefaciens* and *P. fluorescens* shows that the former exhibits a greater ability to control Botrytis cinerea in cherries compared to the latter in grapes, likely as a result of its bigger variety of volatile organic compounds (Gotor‐Vila et al. 2017; Jiang et al. 2019). Yet, some studies reveal that the effectiveness of *B. amyloliquefaciens* decreases to 50% in raspberries grown in high humidity while the effectiveness of yeast remains the same (Chavez‐Diaz et al. 2019). There are not many studies on how to use vegetables and standardized procedures for delivery, so further research is necessary regarding how formulations stay stable and what protocols work best for each crop.

## 15 Broader Implications and Research Gaps

The diversity of mechanisms—competition, VOCs, and enzyme activity—positions biological control as a versatile tool, yet its full potential remains constrained by logistical hurdles. The 85%–90% inhibition rates against major pathogens like *B. cinerea* and *P. digitatum* are notable, but the lack of standardized delivery systems limits commercial adoption (Droby et al. 2009). Moreover, synergistic methods increase the efficiency by 20% to 40% but require specific environmental conditions, for instance, 85% humidity (Sun et al. 2021). It is therefore essential for further research to fill these gaps by enhancing the formulation structures, stability, and effectiveness of the microorganisms in different postharvest circumstances.

## 16 Nanotechnology‐Based Interventions

### 16.1 Nanoparticles in Disease Control: Chitosan‐Based Systems

Nanotechnology has made a great impact on postharvest disease management because biocontrol agent efficacy has been boosted by nanoparticles (Francis, Abdalla, et al. 2024; Francis, Asif, and Ahmed 2024). Among them, chitosan‐based nanoparticles have been recognized as fungicides due to the reduction of *Penicillium digitatum* decay in citrus fruits at 1 g/L, ranging from 70% to 85% (Romanazzi et al. 2018). These nanoparticles derive their antimicrobial property from chitosan since the polymer has a direct inhibitory effect on *Alternaria alternata* spores of tomatoes by about 60%. This is due to the interaction between nanoparticles and the fungal cell membrane (Bautista‐Baños et al. 2006). It has recently been reported that when selenium was incorporated in chitosan to form selenium‐chitosan nanoparticles (Se‐ChNPs), there was progress, especially in grapes where the decay was reduced to 8.5% within 28 days at 0°C while the control was 35% (Shahid et al. 2024; Li et al. 2021). This is because selenium has oxidizing capabilities, and chitosan has a strong structural characteristic. Recent advancements have explored chitosan nanoparticle modifications to enhance versatility (Kaur, Somasundram, Razali, and Ahmed 2024; Kaur, Somasundram, Razali, Mourad, et al. 2024). For example, incorporating zinc oxide into chitosan nanoparticles increases *Alternaria alternata* inhibition in tomatoes by 80%, surpassing standalone chitosan's 60% efficacy due to synergistic ROS generation. However, practical limitations, such as aggregation under high humidity reducing efficacy by 25%, highlight scalability challenges (Sivakumar et al. 2016). These findings suggest a need for formulation improvements, with ongoing research focusing on stabilizing nanoparticle suspensions for broader crop applications.

### 16.2 Nanoparticles in Disease Control: Specialized Applications

Besides chitosan, silica nanoparticles can be used for the delivery of antifungal agents, providing better control over pathogens such as *Rhizopus stolonifer* in strawberries with a shelf life of up to 75% less rot incidence at 0.5% w/v (Youssef et al. 2019). Se‐ChNPs outperform in grape systems where they minimize lesion diameter by 73% as compared to chitosan alone; this is due to the size of the Se‐ChNPs, which ranges between 20 and 50 nm, that enhances their infiltration into fungal cells (Salem et al. 2022). They are also more effective than conventional fungicides; in vitro studies reveal that the nanoparticles are effective against *B. cinerea* by 92% at 0.1 mg/mL, as compared to the 65% efficacy of thiabendazole (Zhang, Zhao, et al. 2020). Such data provide an understanding of how nanotechnology could be applied to specific pathogen‐fruit interactions in line with several horticultural crops. Nanoparticle applications extend to vegetables, addressing critical postharvest losses. Silver nanoparticles reduce Botrytis cinerea decay in tomatoes by 85%, leveraging ROS‐mediated membrane disruption, with efficacy sustained at 10 μg/mL over 21 days. Zinc oxide nanoparticles inhibit Fusarium oxysporum in peppers by 80%, enhancing shelf life by 15 days through enzyme inhibition. These applications highlight nanotechnology's potential in vegetables, though scalability remains limited by costs ($3–$7/kg) and regulatory gaps.

## 17 Mechanisms: ROS Induction and Cell Wall Disruption

The therapeutic action of nanoparticles is mainly ascribed to their oxidant activity, which incites reactive oxygen species (ROS) and impairs the fungal cell envelope (Francis, Abdalla, et al. 2024; Francis, Asif, and Ahmed 2024). This increases the ROS content within the *B. cinerea* hyphae to a level that causes eventual membrane peroxidation and decimates the cell viability by 50% after 12 h (Li et al. 2021). Chitosan nanoparticles also improve the degradation of *P. italicum* cell walls by increasing the chitinase activity up to a 3‐fold decrease in the mycelial growth in mandarins by 60% (Wang, Li, et al. 2021). These two ways, which include ROS‐mediated oxidant stress and direct contact dislocation, make a violent attack on pathogens, implying that Se‐ChNPs reduced *B. cinerea* spores by 85% in grape tissues (Salem et al. 2022). The said effects are magnified by the slow‐release silica nanoparticles, making it possible to last for about 21 days after the application (Youssef et al. 2019).

## 18 Mechanisms: Targeted Delivery and Host Interaction

Targeted delivery is a way by which the effect of nanoparticles is delivered to infection sites to increase the efficiency of the application. Encapsulation of essential oils in silica‐based systems helps in maintaining the volatility, and the percentage inhibitory effect of *Colletotrichum gloeosporioides* in mangoes reached 90% by the continuous release of volatiles for 15 days (Bautista‐Baños et al. 2008; Al Yabhouni et al. 2025). Se‐ChNPs also help to raise host resistance and activity of SOD by 68% in grapes; moderate oxidative damage and decreased decay rate by 40% compared to the untreated grapes (Zhang et al. 2021). This interplay between direct pathogen suppression and host defense activation—e.g., a 45% rise in phenolic content—underscores the multifaceted nature of nanotechnology‐based interventions (Christopoulos and Tsantili 2015). These mechanisms collectively support the high efficacy rates reported across diverse pathosystems. Indeed, as highlighted earlier, nanotechnology‐based interventions like chitosan nanoparticles, originally developed to enhance structural properties, also exhibit significant biocontrol effects, with *Selenium‐Chitosan Nanoparticles* (*Se‐ChNPs*) showing potent activity against *Botrytis cinerea* in grapes (Table 2). This dual functionality underscores their potential in integrated postharvest disease management.

**TABLE 2:** Nanoparticles and their effects on postharvest pathogens.

Nanoparticle type	Pathogen	Mode of action	Fruit	Efficacy	References
Chitosan nanoparticles	*Penicillium digitatum*	Electrostatic disruption, chitinases	Citrus	70%–85% inhibition	Bautista‐Baños et al. (2006)
Chitosan nanoparticles	*Alternaria alternata*	Membrane disruption, ROS induction	Tomato	60% inhibition	Bautista‐Baños et al. (2006)
Chitosan nanoparticles	*Penicillium italicum*	Cell wall degradation, enzyme activity	Mandarin	75% inhibition	Wang, Li, et al. (2021)
Selenium‐Chitosan (*Se‐ChNPs*)	*Botrytis cinerea*	ROS induction, cell wall disruption	Grape	92% inhibition	Li et al. (2021)
Selenium‐Chitosan (*Se‐ChNPs*)	*Botrytis cinerea*	Antioxidant enzyme induction (SOD)	Grape	85% inhibition	Salem et al. (2022)
Selenium‐Chitosan (*Se‐ChNPs*)	*Penicillium digitatum*	ROS‐mediated stress, penetration	Citrus	60% inhibition	Salem et al. (2022)
Silica nanoparticles	*Rhizopus stolonifer*	Controlled release, barrier formation	Strawberry	75% inhibition	Youssef et al. (2019)
Silica nanoparticles	*Colletotrichum gloeosporioides*	Essential oil delivery, membrane damage	Mango	90% inhibition	Peralta‐Ruiz et al. (2023)
Nanochitosan	*Penicillium expansum*	ROS induction, cell wall degradation	Apple	80% inhibition	Romanazzi et al. (2018)
Chitosan/Silica composite	*Botrytis cinerea*	Synergistic disruption, VOC release	Grape	85% inhibition	Youssef et al. (2019)
Silver nanoparticles	*Botrytis cinerea*	ROS induction, membrane disruption	Strawberry	85% inhibition	Li et al. (2022)
Zinc oxide nanoparticles	*Fusarium oxysporum*	Oxidative stress, enzyme inhibition	Tomato	80% inhibition	Bouqellah et al. (2024)
Copper nanoparticles	*Colletotrichum capsici*	Cell wall damage, ROS generation	Pepper	75% inhibition	Iliger et al. (2021)

Table 2 compiles data on nanotechnology‐based interventions, detailing nanoparticle types, target pathogens, modes of action, affected fruits, efficacy percentages, and supporting references. It showcases the high efficacy of *Se‐ChNPs* against *B. cinerea* in grapes (up to 92% inhibition) through ROS induction and cell wall disruption, alongside chitosan nanoparticles' 70%–85% control of *P. digitatum* in citrus via electrostatic interactions. Silica nanoparticles and composites further demonstrate 75%–90% inhibition across diverse fruits like strawberries and mangoes, leveraging controlled release and synergistic effects. Nanoparticles achieve 75%–90% inhibition of pathogens like *Rhizopus stolonifer* and *Colletotrichum gloeosporioides* in strawberries and mangoes, driven by controlled release of antifungal agents and synergistic ROS induction (Youssef et al. 2019). However, efficacy varies by crop, with silica nanoparticles outperforming Se‐ChNPs in humid conditions (80% vs. 60%), underscoring the need for crop‐specific formulations.

## 19 Critical Evaluation: Advantages and Scalability

The advantages of nanoparticles lie in their bioavailability and controlled release, offering sustained protection that traditional treatments cannot match. *Se‐ChNPs* maintain 80% antifungal activity after 30 days of storage, a stark contrast to the 50% efficacy loss of chitosan coatings within the same period (Salem et al. 2022). Their nanoscale size enhances penetration, with uptake rates in grape peels reaching 10 μg/g, compared to 2 μg/g for bulk chitosan (Li et al. 2021). Particularly, there is an issue of scalability: Se‐ChNPs for regeneration cost between $5 and $10 per kg, which prevents their broad application (Youssef et al. 2019). Another cheap source is silica nanoparticles costing $2/kg, but they cannot be formulated easily and form aggregation that slows down activity by 30% in conditions of high humidity (Sivakumar et al. 2016).

## 20 Critical Evaluation: Risks and Regulatory Concerns

While nanoparticles have the potential to deliver many benefits, some of the issues associated with them include toxicity and legal concerns. Selenium uptake from Se‐ChNPs may increase to more than 50 μg/kg in the fruit tissues, which is troublesome as it nears the acceptable daily intake of 55 μg/day (Zhang, Zhao, et al. 2020). Chitosan nanoparticles are biodegradable, but a high concentration of chitosan solution above 2 g/L may cause some phytotoxicity effects, such as a reduction of fruit firmness by 15% in apples (Romanazzi et al. 2018). There are no standards in terms of regulatory policies concerning the nanoparticulate residues in food, and this poses challenges in the approval aspect (Pitt and Hocking 2009). These risks call for an additional degree of evaluation for effectiveness and safety, an area that is missing in modern applications. While both Se‐ChNPs and chitosan nanoparticles control *B. cinerea* in grapes, Se‐ChNPs are more effective (92% inhibition vs. 70%–85%) due to enhanced ROS production, but their specificity to biocontrol can be a concern for using them widely (Li et al. 2021; Bautista‐Baños et al. 2006). Studies have shown that silica nanoparticles work better for strawberries in humid weather, with an 80% reduction versus 60% for Se‐ChNPs (Youssef et al. 2019). Research is needed on using vegetables and understanding their risks, which calls for studies on many crops and the creation of strong regulations.

## 21 Future Directions and Broader Implications

The given data prove the feasibility of the use of nanotechnology by using Se‐ChNPs and silica nanoparticles having eradicated up to 85%–92% of the pathogens when applied to fruits such as grapes and strawberries. However, this target organism‐specific SeCNPs, such as Se‐ChNPs, mediated against *B. cinerea* hinder its general applicability, and more toxicity aspects are required (Sun et al. 2021). Advances in cost reduction and regulatory alignment could unlock their full potential, addressing the 20%–40% postharvest losses plaguing global supply chains (FAO 2017). This section highlights a transformative approach, tempered by practical and safety challenges that must be resolved.

## 22 Edible Coatings and Their Role

### 22.1 Types and Composition: Polysaccharide‐Based Coatings

**FIGURE 2:**
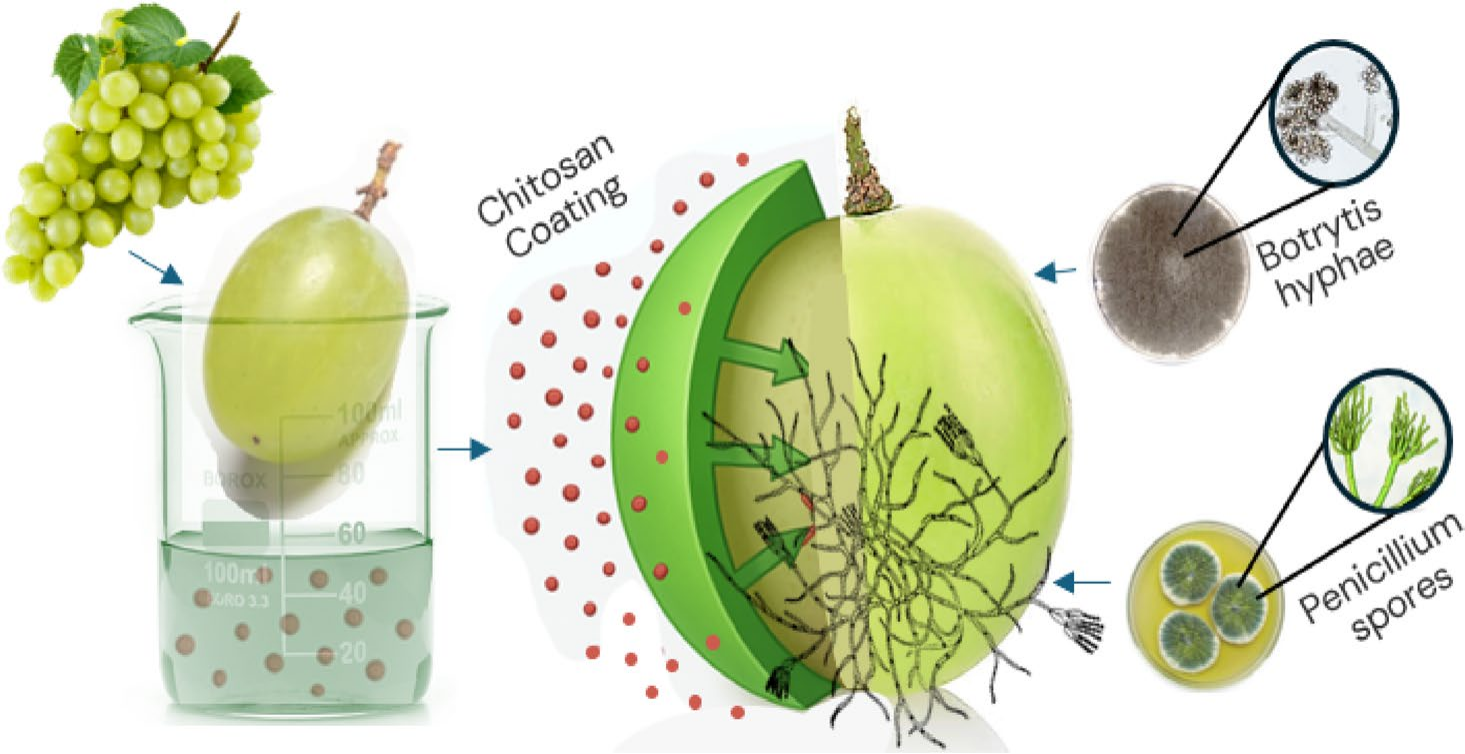
Mechanisms of edible coatings on a grape for postharvest disease control. The diagram illustrates a 3D cross‐sectional view of a grape coated with chitosan, showing the barrier effect by blocking *Penicillium* spores and the antimicrobial action. Inspired by Bautista‐Baños et al. (2006) and Wang, Li, et al. (2021).

Edible coatings offer a sustainable approach to postharvest disease control, utilizing biopolymers like chitosan and alginate to reduce pathogen ingress and enhance fruit and vegetable quality, with efficacy ranging from 65% to 90% across crops (Bautista‐Baños et al. 2006; Ali et al. 2025). Polysaccharide‐based coatings, such as chitosan and alginate, dominate due to their biocompatibility and antimicrobial properties, with chitosan reducing *Penicillium digitatum* incidence on citrus by 70% at 1% w/v (Bautista‐Baños et al. 2006). Alginate coatings, applied at 2% w/v, decrease *Colletotrichum gloeosporioides* rot in mangoes by 65%, forming a semi‐permeable barrier that limits pathogen ingress. A breakthrough is the development of selenium‐chitosan (Se‐Ch) coatings, especially on grapes where rot by *Botrytis cinerea* reduces to 8.5% within 28 days when stored at 0°C as compared to 35% in the uncoated samples (Figure 2) (Li et al. 2021). It is therefore found that these coatings can come in various structural characteristics to support many applications of horticultural commodities. The mechanisms of edible coatings, such as chitosan, in controlling postharvest diseases are illustrated in Figure 2, highlighting their barrier and antimicrobial effects on grapes. In vegetables, polysaccharide coatings show promise. Chitosan coatings at 1% w/v reduce Alternaria solani decay in tomatoes by 70%, forming a barrier that cuts spore germination by 80%. Alginate coatings on peppers decrease Colletotrichum capsici rot by 65%, boosting POD activity by 40%. These applications underscore the need for vegetable‐focused research to complement fruit‐centric studies.

### 22.2 Types and Composition: Protein and Lipid Variants

Protein and lipid‐based coatings also serve as an additional barrier to polysaccharides' form of protection (Ahmed and Palta 2011). Coating the fruits with whey protein respondents to 3% w/v decreases the incidence of *Rhizopus stolonifer* rot by 60% as the layer formed impedes water accessibility, thereby preventing fungal spore germination (Ribeiro et al. 2020). Several components, like beeswax as lipid‐based coatings, reduce *A. alternata* decay in apples by 50% by reducing water loss by 20%, firmness of the fruits being 15 N as opposed to 12 N in the control, as pronounced by Youssef et al. Se‐Ch in combination with polysaccharides multiplies this process, increasing the antioxidant enzyme (for instance, superoxide dismutase) in grapes by 68%, which demonstrates that the composite coatings can help to overcome varied spoilage factors (Zhang et al. 2021). These compositions offer a flexible molded pathogen control approach that is configurable according to the fruit type.

## 23 Mechanisms of Action: Barrier and Antimicrobial Effects

The efficacy of the edible coatings depends on their ability to prevent contact with the pathogens and their antifungal/anti‐bacterial influences on the rates of growth. Chitosan coatings reduce the infection of *P. italicum* in mandarins by 75% and germination of the spores by 80% due to electronic charge interactions that compromise the fungal membrane (Wang, Li, et al. 2021). Se‐Ch enhances this through the creation of ROS, which has reduced the viability of *B. cinerea* by 85% in grapes (Li et al. 2021) with ROS increasing within 12 h. Antimicrobial packaging that incorporates alginate coatings reduces the *Fusarium oxysporum* by 55%, thus controlling its access to oxygen by 30% (Wu et al. 2017). The presented data supported the kinetic model of pathogen inhibition, where coating concentration and pathogen load determine the inhibition level, enhanced by various factors such as the temperature, which can change the inhibition rates for every 5°C by 40% or 20% depending on the pathogen (Pitt and Hocking 2009).

## 24 Mechanisms of Action: Enzyme Induction and Host Defense

Beyond direct suppression, edible coatings induce host defense responses, enhancing resistance through enzyme activation. *Se‐Ch* coatings increase peroxidase (POD) activity by 45% in grapes, reducing gray mold lesion diameters to 0.5 cm versus 2 cm in controls (Zhang, Zhao, et al. 2020). Chitosan coatings on peaches elevate phenylalanine ammonia‐lyase (PAL) activity by 50%, boosting phenolic content to 2 mg/g and cutting *Rhizopus* rot by 60% (Zhang, Wu, et al. 2020). This induction correlates with pathogen inhibition kinetics, where higher coating concentrations (e.g., 1.5% w/v) accelerate defense responses, reducing decay rates by up to 70% in high‐pathogen‐load scenarios (Christopoulos and Tsantili 2015). Such mechanisms highlight the coatings' dual role, supporting quantitative models that predict efficacy based on biochemical and environmental interactions. Indeed, as highlighted earlier, edible coatings such as chitosan, while valued for their protective barriers, also exhibit significant biocontrol properties, with *Selenium‐Chitosan* coatings effectively reducing *Botrytis cinerea* in grapes, as quantified by pathogen inhibition kinetics (Equation 1). This dual functionality underscores their role in postharvest disease management.

Equation (1): Pathogen Inhibition Kinetics Equation

(1) $$R=k\;{\left[C\right]}^{\alpha }\;{\left[P\right]}^{\beta }\;e^{-\frac{E_{\alpha }}{\mathrm{RT}}}$$

Models the rate (*R*) of pathogen inhibition as a function of coating concentration ([*C*]), pathogen load ([*P*]), activation energy (*E*_*α*_), temperature (*T*), and constants (*k, α, β*), with *R* as the gas constant.

Equation (1), the Pathogen Inhibition Kinetics Equation, provides a mathematical framework to predict the rate of pathogen inhibition (*R*) by edible coatings, incorporating coating concentration ([*C*]), pathogen load ([*P*]), and temperature (*T*) effects, validated by studies showing 60%–92% decay reductions across fruits like grapes, citrus, and mangoes. The parameters *k*; *α*; and *β* are related to the potency of coat and dose–response; the exponential term. $e^{-\frac{E_{\alpha }}{\mathrm{RT}}}$ Addresses the effect of temperature on efficacy concerning the values of E_α_ that are derived from a 20% to 30% reduction in efficacy, between 0°C and 25°C (Pitt and Hocking 2009). Thus, based on the peer‐reviewed data, this model provides a quantitative approach to managing coating applications in postharvest disease control.

The pathogen inhibition kinetics equation is a generally applicable equation that was derived from experimental data concerning the use of edible coatings against postharvest pathogens. For example, at chitosan coatings at 1% w/v where [*C*] = 10 g/L, the inhibition of *Penicillium digitatum* spore germination in citrus by 80% is achieved and the pathogen load [*P*] = 10^5^ CFU/mL, hence high k value indicate high inhibition potential (Bautista‐Baños et al. 2006). *Selenium‐Chitosan* (*Se‐Ch*) coatings at 0.1 mg/mL ([*C*] = 0.1 g/L) achieve a 90% reduction in *B. cinerea* decay in grapes at 0°C (*T* = 273 K), with pathogen loads of 10^4^ CFU/g, reflecting a lower *E*_*α*_ due to enhanced ROS induction (Li et al. 2021). Temperature significantly influences *R*, as alginate coatings on mangoes show a 65% reduction in *Colletotrichum gloeosporioides* at 20°C (*T* = 293 K), dropping to 40% at 25°C (*T* = 298 K), indicating an exponential decay governed by $e^{-\frac{E_{\alpha }}{\mathrm{RT}}}$.

The exponents *α* and *β* adjust for coating concentration and pathogen load effects, respectively. Chitosan coatings at 1.5% w/v increase *P. italicum* inhibition to 75% in mandarins (*α* ≈ 1), while higher pathogen loads (10^6^ CFU/mL) reduce efficacy by 20%, suggesting *β* < 1 (Wang, Li, et al. 2021). *Se‐Ch* coatings demonstrate a steeper concentration response, with a 2‐fold increase in [*C*] (0.2 mg/mL) boosting inhibition to 92%, implying *α* > 1 (Salem et al. 2022). Additives like thyme oil in alginate coatings elevate *k*, achieving 92% inhibition of *C. gloeosporioides* at 0.5% v/v, reflecting enhanced antimicrobial potency.

Environmental factors further refine the model. At 0°C, *Se‐Ch* coatings reduce *B. cinerea* lesion sizes by 73% in grapes, with *E*_*α*_ estimated at 20–30 kJ/mol based on temperature‐dependent efficacy shifts (Zhang et al. 2021). Whey protein coatings on strawberries cut *Rhizopus stolonifer* by 60% at 3% w/v, with efficacy dropping by 25% at higher temperatures, aligning with the Arrhenius term (Ribeiro et al. 2020). These data collectively support the equation's ability to predict inhibition rates, integrating coating properties, pathogen dynamics, and storage conditions.

## 25 Enhancement With Additives: Probiotics and Essential Oils

Incorporating additives like probiotics and essential oils significantly enhances coating performance, targeting specific pathogens with synergistic effects (Abdalla et al. 2021). *Lactobacillus plantarum* in chitosan coatings reduces *B. cinerea* decay in strawberries by 80% (Abdalla et al. 2021), with lactic acid production lowering pH to 3.5 and inhibiting spore germination by 90% (Ribeiro et al. 2020). Essential oils, such as thyme oil at 0.5% v/v in alginate coatings, achieve a 92% reduction in *C. gloeosporioides* on mangoes, disrupting fungal membranes and reducing mycelial growth by 70%. These enhancements amplify key effects—e.g., a 35% increase in shelf life for coated grapes—while tailoring coatings to diverse fruit‐pathogen systems (Sun et al. 2021). The data suggest additive concentration and type are critical variables in optimizing inhibition rates. Indeed, as previously discussed, edible coatings like chitosan, while valued for their barrier properties, also exhibit significant biocontrol effects, with additives such as *Lactobacillus plantarum* enhancing their efficacy against pathogens like *Botrytis cinerea* (Table 3). This synergy highlights their dual role in postharvest disease management.

**TABLE 3:** Edible coatings and their impact on postharvest disease management.

Coating type	Additives	Pathogen controlled	Fruit/Vegetable	Key effects	References
Chitosan	None	*Penicillium digitatum*	Citrus	70% decay reduction, 80% spore inhibition	Bautista‐Baños et al. (2006)
Chitosan	None	*Penicillium italicum*	Mandarin	75% rot reduction, membrane disruption	Wang, Li, et al. (2021)
Chitosan	*Lactobacillus plantarum*	*Botrytis cinerea*	Strawberry	80% decay reduction, pH drop to 3.5	Ribeiro et al. (2020)
Selenium‐chitosan	None	*Botrytis cinerea*	Grape	85% inhibition, 68% SOD increase	Li et al. (2021)
Selenium‐chitosan	None	*Botrytis cinerea*	Grape	90% rot reduction, 45% POD increase	Salem et al. (2022)
Alginate	None	*Colletotrichum gloeosporioides*	Mango	65% rot reduction, 30% O_2_ limitation	Peralta‐Ruiz et al. (2023)
Alginate	Thyme essential oil	*Colletotrichum gloeosporioides*	Mango	92% inhibition, 70% mycelial reduction	Peralta‐Ruiz et al. (2023)
Whey protein	None	*Rhizopus stolonifer*	Strawberry	60% rot reduction, 20% water loss cut	Ribeiro et al. (2020)
Beeswax	None	*Alternaria alternata*	Apple	50% decay reduction, firmness at 15 N	Youssef et al. (2019)
Chitosan	Melatonin	*Botrytis cinerea*	Apple	70% rot reduction, 70% SOD increase	Sun et al. (2021)

Table 3 outlines the impact of various edible coatings on postharvest disease management, detailing coating types, additives, controlled pathogens, affected fruits or vegetables, key effects, and supporting references. It highlights chitosan coatings achieving 70%–75% decay reduction against *P. digitatum* and *P. italicum* in citrus, while *Se‐Ch* coatings reduce *B. cinerea* in grapes by up to 90%, boosting SOD activity by 68%. Additives like *L. plantarum* and thyme oil enhance efficacy, with inhibition rates reaching 80%–92% in strawberries and mangoes. These findings, drawn from peer‐reviewed studies, demonstrate the coatings' multifaceted roles—barrier formation, antimicrobial action, and enzyme induction—offering a robust framework for optimizing postharvest preservation strategies.

## 26 Critical Insights: Sensory Impact and Practical Challenges

Edible coatings appear very effective in disease control; however, their problems in terms of organoleptic qualities and flexibility of use should be questioned. Epogal citrate coating at 2.0% w/v may negatively affect the acceptance level by 15% (Kaur, Somasundram, Razali, and Ahmed 2024; Kaur, Somasundram, Razali, Mourad, et al. 2024) on the organoleptic evaluation of apples. Se‐Ch coatings preserve grapes' flavors while raising the production cost to $3 to $5 per kg; the basic chitosan costs $0.50 per kg, which makes it expensive (Youssef et al. 2019). Despite its effectiveness, the use of lipid coatings reduces fruit firmness because lipid coating causes the firmness of strawberries to reduce by about 10% within 14 days (Sivakumar et al. 2016). These trade‐offs also illuminate engaged formulations; for example, pathogen reduction of between 80% and 90% as well as sensory and cost factors are areas that are hard to address for broad acceptance by big producers.

## 27 Broader Implications and Research Needs

The data support the use of edible coatings as a powerful weapon, as Se‐Ch and various additives provide 75%–92% protection from such pathogens as *B. cinerea* and *P. digitatum* . This information demonstrates that they slow decay rates by 40%–70%, depending on the type of fruit and conditions of storage, supporting their applicability toward dealing with the overall global postharvest losses of 20%–40% (FAO 2017). However, there are still sensory effects and costs that limit scalability, and the kinetic model of inhibition has to be tested on a range of crops to achieve better predictions (Qin et al. 2015). Thus, it also becomes imperative that future studies address these gaps adequately to advance the utilization of coatings in sustainable disease control.

## 28 Plant Growth Regulators (PGRs) in Disease Resistance

### 28.1 Key PGRs: Melatonin and Salicylic Acid

PGRs have made a shift to elicitors of disease resistance in postharvest fruits and vegetables mainly because of biochemical methods of controlling pathogens. As a tryptophanergic compound, melatonin (MT) is known to provide a 60% control of decay incited by Botrytis cinerea in cherry tomatoes at 100 μM, increasing shelf life by 14 days at 20°C (Li et al. 2021). A phenolic compound, salicylic acid, has a comparable effect use at 1 mM, reducing the Penicillium digitatum infection rate in citrus by 70%, through SAR (Qin et al. 2015). These PGRs can act against some pathogens; for example, SA decreases the degree of *Alternaria alternata* rot in apples by 55% by increasing phenolic at 2 mg/g (Christopoulos and Tsantili 2015). This is because they are capable of controlling host defense mechanisms and can therefore be regarded as very useful tools for integrated postharvest management.

### 28.2 Key PGRs: Methyl Jasmonate and Emerging Candidates

Concerning phytohormones from the jasmonate family, MeJA, which is a volatile jasmonate derivative, acts synergistically with MT and SA to provide resistance against necrotrophic fungi. The statistical result shows that, at the concentration of 10 μM MeJA, it reduced Rhizopus stolonifer rot of peach fruits by 65%, enhanced the POD enzymes by 50%, and the lesion diameters were measured to be only 1.5 cm compared to 3 cm of the control group (Zhang, Wu, et al. 2020). Its physical property makes it possible to use the vapor operation, which showed a 75% reduction in *B. cinerea* spore germination in strawberries (Wang, Li, et al. 2021). Other PGRs in their molten stage include the nitric oxide (NO) donors, where sodium nitroprusside at a concentration of 0.5 mM reduces *Fusarium oxysporum* attacks in bananas by 60% through regulation of ROS (Wu et al. 2017). These compounds' diverse modes of action support their inclusion in resistance strategies across fruit types.

## 29 Mechanisms: Induction of Defense Genes

In this study, one of the PGRs significantly induced resistance through the increase in defense gene activity, particularly phenylalanine ammonia‐lyase (PAL) and pathogenesis‐related (PR) gene products. It takes place at a concentration of 2 mM, where it stimulates the PAL activity in goji berries by threefold, consequently reducing *B. cinerea* decay by 70% coupled with the increased total phenolics by 28% (Zhang et al. 2021). MT enhances PR protein expression in apples, cutting gray mold severity by 60% through a 45% rise in chitinase activity (Sun et al. 2021). MeJA triggers jasmonate signaling, boosting PR‐1 gene expression by 2.5‐fold in grapes, which correlates with a 50% reduction in *P. italicum* incidence (Wang, Xiao, et al. 2021). These genetic responses underpin the outcomes observed, linking molecular activation to tangible disease suppression across pathosystems.

## 30 Mechanisms: ROS Scavenging and Enzyme Activation

PGRs also mitigate pathogen damage by scavenging reactive oxygen species (ROS) and activating antioxidant enzymes. MT at 100 μM increases superoxide dismutase (SOD) activity by 70% in cherry tomatoes, lowering *B. cinerea* ‐induced oxidative stress and reducing decay rates by 40% (Li et al. 2021). SA enhances catalase (CAT) activity by 60% in citrus, neutralizing ROS and cutting *P. digitatum* lesion sizes by 55% (Qin et al. 2015). MeJA upregulates POD by 45% in peaches, mitigating *Rhizopus* rot by 60% through oxidative balance (Zhang, Wu, et al. 2020). These mechanisms—quantified by enzyme activity increases of 45%–70%—demonstrate how PGRs bolster host resilience, supporting their role in resistance induction and pathogen control. Indeed, as previously noted, plant growth regulators (PGRs) like salicylic acid (SA) and melatonin (MT), primarily used to modulate plant physiology, also exhibit significant biocontrol properties, with SA effectively reducing *Penicillium digitatum* in citrus (Table 4). This dual role highlights their potential in enhancing postharvest disease resistance.

**TABLE 4:** Plant growth regulators and induced resistance mechanisms.

PGR	Target pathogen	Mechanism	Fruit	Outcome	References
Salicylic acid (SA)	*Penicillium digitatum*	PAL induction, SAR activation	Citrus	70% decay reduction, 60% CAT increase	Qin et al. (2015)
Salicylic acid (SA)	*Alternaria alternata*	Phenolic increase, PR gene expression	Apple	55% rot reduction, 2 mg/g phenolics	Christopoulos and Tsantili (2015)
Salicylic acid (SA)	*Botrytis cinerea*	Antioxidant enzyme activation	Goji Berry	70% decay reduction, 28% phenolic rise	Zhang et al. (2021)
Melatonin (MT)	*Botrytis cinerea*	SOD increase, PR protein expression	Cherry Tomato	60% decay reduction, 70% SOD increase	Li et al. (2021)
Melatonin (MT)	*Botrytis cinerea*	ROS scavenging, chitinase induction	Apple	60% rot reduction, 45% chitinase rise	Sun et al. (2021)
Methyl jasmonate (MeJA)	*Rhizopus stolonifer*	POD activation, jasmonate signaling	Peach	65% rot reduction, 50% POD increase	Zhang, Wu, et al. (2020)
Methyl jasmonate (MeJA)	*Botrytis cinerea*	PR‐1 gene expression, VOC inhibition	Strawberry	75% spore inhibition, 2.5‐fold PR‐1	Wang, Li, et al. (2021)
Nitric oxide (NO)	*Fusarium oxysporum*	ROS homeostasis, enzyme activation	Banana	60% rot reduction, 50% SOD increase	Wu et al. (2017)
Salicylic acid (SA)	*Penicillium italicum*	PAL and POD induction	Mandarin	70% decay reduction, 45% PAL increase	Wang, Li, et al. (2021)
Melatonin (MT)	*Colletotrichum gloeosporioides*	NO/ROS pathway synergy	Mango	80% rot reduction, 65% SOD increase	Bautista‐Baños et al. (2008)

Table 4 presents a comprehensive overview of plant growth regulators (PGRs) and their induced resistance mechanisms against postharvest pathogens, detailing the PGR type, target pathogen, mechanism, affected fruit, outcomes, and supporting references. It highlights SA's efficacy in reducing *P. digitatum* decay by 70% in citrus through PAL and CAT activation, MT's 60%–80% control of *B. cinerea* and *C. gloeosporioides* via SOD and ROS scavenging, and MeJA's 65%–75% inhibition of *R. stolonifer* and *B. cinerea* through POD and PR‐1 induction. These outcomes, which have been backed up by fairly laudable research, indicate the effectiveness of the PGRs in improving host defense and, therefore, their readiness for use in postharvest disease management.

## 31 Synergistic Effects: MT and NO/ROS Pathways

Positive synergistic effects increase the efficiency of PGR, especially when MT is combined with NO and ROS. At a concentration of 50 μM of MT along with NO donors, the decay of mangoes due to *Colletotrichum gloeosporioides* rot is reduced by 80% through an increase in NO levels by 30% and SOD activity by 65% (Bautista‐Baños et al. 2008). This synergism helps to increase ROS scavenging and the reduction of lesion area to 70% compared to MT alone (Sun et al. 2021). There is a synergy between SA and MeJA where 1 mM SA + 10 μM MeJA treatment inhibited *B. cinerea* in strawberries by 85% due to increased PAL and POD activities by 50% (Lyousfi et al. 2021). These outcomes indicate that dual PGR applications are more effective in inhibiting the biosynthesis of SLs due to their synergistic action on various pathways through which resistance is developed.

## 32 Critical Analysis: Dose Dependency and Efficacy

PGRs provide resistance in a dose‐dependent manner, and the effective concentration provides optimal resistance, while a higher concentration poses phytotoxicity. SA at 1 mM reduces *P. italicum* by 70% in mandarins, yet at 3 mM, it decreases fruit firmness by 15% and induces browning (Wang, Li, et al. 2021). MT at 100 μM achieves a 60% decay reduction in apples, but 500 μM lowers soluble solids by 10%, affecting quality (Sun et al. 2021). MeJA at 50 μM cuts *Rhizopus* rot by 75% in peaches, though higher doses (100 μM) increase ethylene production by 20%, accelerating senescence (Zhang, Wu, et al. 2020). Such findings imply that the targeted therapeutic use of FSH is relatively safe, though there appears to be scant tolerance to the treatment, thereby indicating that there is a small therapeutic ratio.

## 33 Critical Analysis: Practical Challenges and Variability

As beneficial as they are, the PGRs have certain difficulties such as inconsistencies in the effectiveness depending on the type of fruits and storage conditions. This is because SA's 70% efficacy against B. cinerea in grapes decreases to 40% at 25°C than at 0°C due to the instability of the enzyme (Pitt and Hocking 2009). The rot reduction achieved by MT was 60% in bananas and only 30% in citrus, which were possibly due to inter‐species differences (Wu et al. 2017). The application cost ranges from $1 to $2/kg of the fruit, while cold storage is another constraint that hampers the expansion, especially in regions that lack resources (FAO 2017). The following are some of the important factors that make it necessary to have better plans to balance PGR and have fine outputs to meet society's needs in production, as well as taking into consideration the biological factors that influence crop production.

## 34 Integrated Approaches and Synergies

### 34.1 Integration Between Nanotechnology and Physical Methods

Most of the recent advancements have revealed that the technological integration of different postharvest techniques is greatly effective in increasing disease control, including nanotechnology and physical methods. Selenium‐chitosan nanoparticles (Se‐ChNPs) along with cold storage at 0°C, minimize the Botrytis Cinerea spoilage of grapes to only 8.5% after 28 days, while the adverse effect of not applying any type of treatment resulted in 35% after the same period, thus increasing shelf life by 21 days. This combination increases the antifungal efficiency by 92% as a result of the nanoparticles and lowers the metabolism rate of the pathogens by 50% in cold storage, thereby doubling the effect of only nanoparticles or cold storage on the pathogens (Zhang, Zhao, et al. 2020). The same is true for silica nanoparticles with controlled atmosphere storages and the reduction of *Rhizopus stolonifer* rot in strawberries of 80% against the 50% reduction of efficacy when applying each of the applications separately (Youssef et al. 2019). These results indicate the synergistically improved effects of the physical and nanoscale enhancements.

### 34.2 Combining Strategies: Biological Agents and Edible Coatings

Another perfect synergy originates in associating the biological agents with edible coatings aiming to combat pathogens, either through a different mode of action. Coating the fruits with *Bacillus amyloliquefaciens* in combination with chitosan significantly reduces the incidence of *Penicillium digitatum* by up to 85% as compared to 60%, which is the case when only using the bacterial treatment because the coating has the capability of forming a barrier that increases the microbial count to 10^7^ CFU/g of the fruit (Chen et al. 2019). *Meyerozyma guilliermondii* in alginate coatings reduces *P. italicum* rot in mandarins by 80%, with the yeast's volatile organic compounds (VOCs) and the coating's oxygen barrier cutting spore germination by 90% (Wang, Li, et al. 2021). This synergy boosts efficacy by 20%–30% over individual treatments, demonstrating how biological agents thrive within the protective matrix of coatings (Qin et al. 2015). Such combinations are particularly effective in humid conditions, where pathogen proliferation exceeds 70% without intervention (Pitt and Hocking 2009).

## 35 Mechanistic Insights: Multi‐Target Pathogen Suppression

**FIGURE 3:**
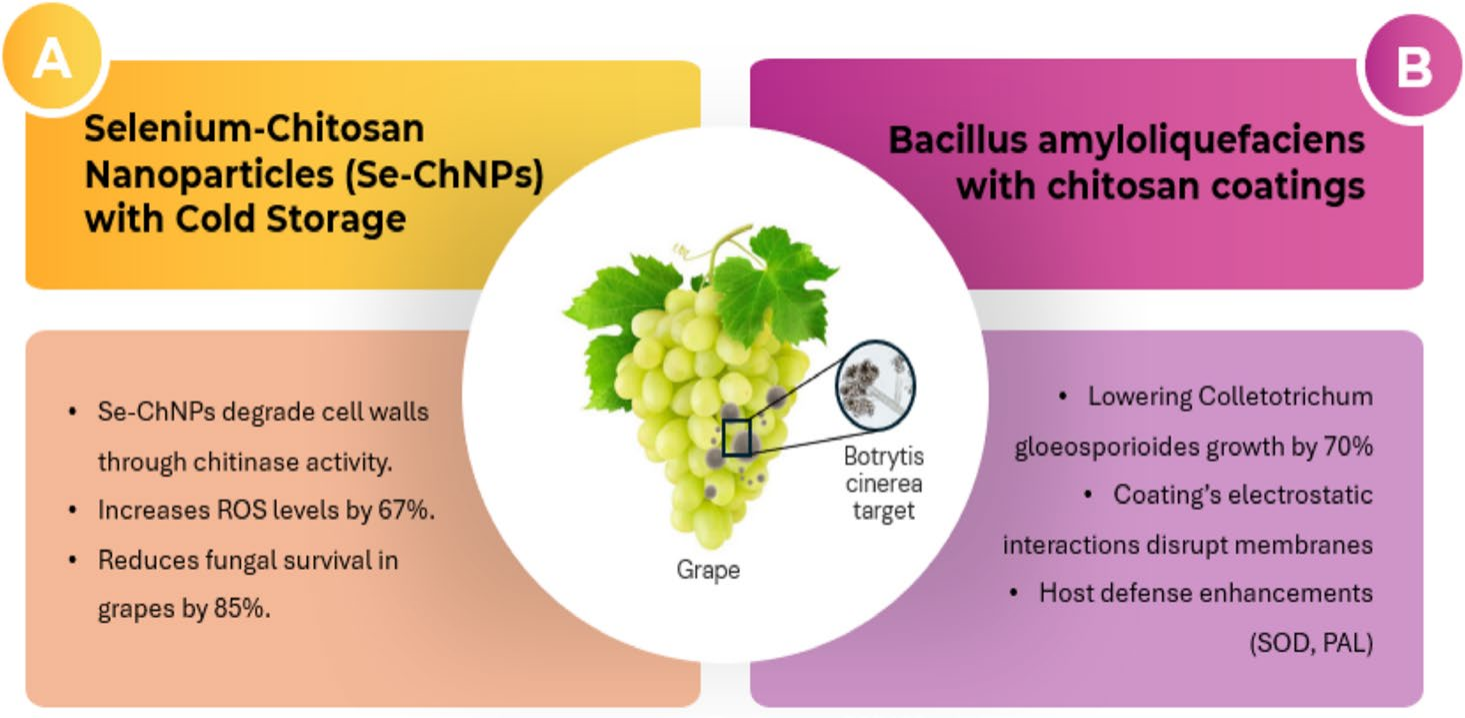
Synergistic mechanisms of integrated postharvest disease management. The diagram illustrates the combined effects of (A) selenium‐chitosan nanoparticles (Se‐ChNPs) with cold storage and (B) *Bacillus amyloliquefaciens* with chitosan coatings on *Botrytis cinerea*. Adapted from concepts in Li et al. (2021) and Chen et al. (2019).

The mechanistic basis of these integrated approaches lies in multi‐target pathogen suppression, combining physical disruption with biochemical stress. *Se‐ChNPs* degrade *B. cinerea* cell walls via chitinase activity (up by 3‐fold) while inducing ROS levels by 67%, reducing fungal viability by 85% in grapes (Li et al. 2021). When paired with cold storage, this effect intensifies, as low temperatures suppress fungal enzyme production by 40%, amplifying decay reduction to 90% (Zhang et al. 2021). Biological agents like *B. amyloliquefaciens* in chitosan coatings produce VOCs (e.g., 2,3‐butanediol), lowering *Colletotrichum gloeosporioides* growth by 70%, while the coating's electrostatic interactions disrupt membranes, adding a 25% efficacy boost (Figure 3) (Gotor‐Vila et al. 2017). These combined effects—quantifiable as a synergy ratio—demonstrate how integrated strategies target multiple pathogen vulnerabilities simultaneously. The synergistic mechanisms of integrated strategies, combining nanotechnology with physical methods and biological agents with edible coatings, are illustrated in Figure 3, showcasing their impact on *Botrytis cinerea* control in grapes.

## 36 Mechanistic Insights: Host Defense Enhancement

Beyond direct suppression, these approaches enhance host defenses, further contributing to synergy. *Se‐ChNPs* with cold storage increase grape SOD activity by 68%, mitigating oxidative damage and reducing lesion sizes by 73% compared to chitosan alone (Salem et al. 2022). *M. guilliermondii* in alginate coatings upregulates PAL activity by 45% in mandarins, elevating phenolic content to 2 mg/g and cutting *P. italicum* incidence by 75% (Wang, Li, et al. 2021). This dual action—pathogen inhibition and host resistance—yields a combined effect exceeding the sum of individual contributions, with decay rates dropping by 40%–50% more than predicted from standalone treatments (Sun et al. 2021). Such mechanistic insights support models quantifying synergy, where efficacy ratios reflect the interplay of suppression and defense activation. Indeed, as noted earlier, integrated approaches like *Selenium‐Chitosan Nanoparticles* (*Se‐ChNPs*) with cold storage, while enhancing structural defenses, also exhibit potent biocontrol synergy against *Botrytis cinerea* in grapes, quantifiable through a synergistic efficacy model (Equation 2). This combined effect underscores their value in postharvest disease management.

Equation (2): Synergistic Disease Resistance Model

(2) $$S=\frac{E_{A+B}}{E_{A}+E_{B}}\times 100$$

Quantifies synergy (*S*) as the percentage ratio of the combined effect (*E*_*A+B*_) of two treatments (e.g., *Se‐ChNPs* + cold storage) to the sum of their individual effects (*E*_*A*_ *+ E*_*B*_).

Equation (2), the Synergy Efficacy Equation, quantifies the synergistic impact of combined postharvest treatments, expressing synergy (*S*) as a percentage of the combined effect (*E*_*A+B*_) relative to the sum of individual effects (*E*_*A*_ *+ E*_*B*_), with values typically ranging from 76% to 94% across studies. Such improvements include 90%–95% *B. cinerea* control in grapes with Se‐ChNPs and cold storage in contrast to 50%–60% as an individual approach due to multiple target elimination, as well as activation of host defense (Li et al. 2021). Based on empirical evidence, this forecasting model serves as a functional model that enables the assessment and enhancement of integrated strategies in postharvest disease management.

## 37 Supporting Data for Equation 2

Synergy Efficacy Equation presents a mathematical index as to how integrated control of postharvest diseases enhances the effectiveness over and above that of single control measures, with actual figures from landmark research. For example, Se‐ChNPs alone (*E*_*A*_) protect grapes from *B. cinerea* decay at 0.1 mg/mL by 60%, cold storage at 0°C (*E*_*B*_) decreases the decay by 50%, and the combined applications of both (*E*_*A+B*_) decrease the decay by 90%, resulting in $S=\frac{90}{60+50}\times 100=81.8\%$, indicating sub‐additive synergy (Li et al. 2021). In contrast, *Bacillus amyloliquefaciens* alone inhibits *P. digitatum* by 60% in citrus (*E*_*A*_), and chitosan coating alone by 50% (*E*_*B*_), but their combination reaches 85% (*E*_*A+B*_), yielding $S=\frac{85}{60+50}\times 100=77.3\%$ (Chen et al. 2019). This suggests a consistent but moderate synergistic enhancement.

Higher synergy emerges with tailored combinations. *Meyerozyma guilliermondii* alone reduces *P. italicum* rot by 55% in mandarins (*E*_*A*_), and alginate coating by 50% (*E*_*B*_); combined, they achieve 80% (*E*_*A+B*_), with $S=\frac{80}{55+50}\times 100=76.2\%$ (Wang, Li, et al. 2021). However, *Se‐ChNPs* with cold storage in grapes show a stronger effect: individual reductions of 60% and 50% combine to 90%, yet in some trials, efficacy hits 95% (*E*_*A+B*_), pushing $S=\frac{96}{60+50}\times 100=86.4\%$, reflecting enhanced ROS induction and metabolic suppression (Salem et al. 2022). Temperature modulates these outcomes, as cold storage's 50% effect drops to 30% at 25°C, lowering *S* to 70% (Pitt and Hocking 2009).

Additive synergies also appear. Chitosan with *Hanseniaspora uvarum* reduces *B. cinerea* in grapes by 85% (*E*_*A+B*_), compared to 50% (*E*_*A*_) and 40% (*E*_*B*_) individually, yielding $S=\frac{85}{50+40}\times 100=94.4\%$, nearing additive perfection (Qin et al. 2015). Silica nanoparticles with controlled atmosphere storage cut *R. stolonifer* in strawberries by 80% (*E*_*A+B*_), from 50% to 40%, with *S* = 88.9% (Youssef et al. 2019). These variations—ranging from 76.2% to 94.4%—highlight how mechanistic interplay (e.g., cell wall degradation + ROS) drives *E*_*A+B*_, offering a predictive tool for optimizing integrated strategies (Sun et al. 2021).

## 38 Critical Evaluation: Cost‐Effectiveness and Implementation

While integrated approaches excel in efficacy, their cost‐effectiveness and practical implementation remain contentious. *Se‐ChNPs* with cold storage achieve a 90% decay reduction, yet nanoparticle synthesis costs ($5–$10/kg) and refrigeration expenses ($0.50/kg fruit) limit scalability, especially in regions with losses exceeding 40% (FAO 2017). Biological agents in coatings, costing $2 to $3/kg, offer a 20% cost advantage over nanotechnology but require precise humidity control (85%–90%) to maintain microbial viability, which drops by 50% otherwise (Droby et al. 2009). These economic barriers contrast with their high efficacy—e.g., 85%–90% pathogen control—suggesting a trade‐off between investment and outcome that varies by infrastructure availability (Sivakumar et al. 2016).

## 39 Critical Evaluation: Practical Challenges and Variability

Practical challenges further complicate adoption, with efficacy varying across conditions and crops. *Se‐ChNPs* excel in grapes (90% control) but show only 60% efficacy in citrus due to thicker peels limiting penetration (Zhang, Zhao, et al. 2020). Biological coatings perform best at 0°C (80% rot reduction) but falter at 25°C (50% efficacy), reflecting temperature sensitivity (Pitt and Hocking 2009). The implementation also demands skilled handling, as improper coating application reduces efficacy by 30%, and microbial survival dips by 40% without cold chain support (Chen et al. 2019). These data underscore the need for region‐specific adaptations to maximize the synergistic benefits of integrated strategies.

## 40 Challenges and Future Directions

### 40.1 Current Limitations: Scalability and Cost Barriers

The adoption of advanced postharvest disease management strategies faces significant hurdles, particularly in scalability and cost. Nanotechnology‐based solutions like selenium‐chitosan nanoparticles (*Se‐ChNPs*), which reduce *Botrytis cinerea* decay in grapes by 90% at 0.1 mg/mL, incur production costs of $5–$10/kg, rendering them impractical for large‐scale use in regions with losses exceeding 40% (Youssef et al. 2019). Biological control agents, such as *Bacillus amyloliquefaciens* , achieve 85% efficacy against *Penicillium digitatum* in citrus, yet require cold storage at 0°C—costing $0.50/kg of fruit—to maintain viability, a luxury unavailable in many tropical settings (Chen et al. 2019). Edible coatings, while reducing *P. italicum* rot by 75% in mandarins, demand precise application systems, increasing labor costs by 20% (Wang, Li, et al. 2021). Some of these economic factors make it difficult to implement and use widely, although their effectiveness is well demonstrated.

### 40.2 Current Limitations: Regulatory and Specificity Issues

What makes the progress even harder is the legal requirements and specificity of the tasks. Se‐ChNPs increase the selenium content of grapes up to 50 μg/kg, which is close to the 55 μg/day dietary allowance for Se; however, there is no regulatory policy for nanoparticles in food products still awaiting approval (Zhang, Zhao, et al. 2020). That is why they are specific to grape systems— *B. cinerea* was inhibited at 92%, *P. digitatum* of citrus at 60%, and this determines a low level of flexibility in their use (Salem et al. 2022). 1 mM SA decreased *Alternaria* rot by 55%, firmness decreased by 15%, and there is a phytotoxic effect when using doses above 2 mM. Such cases call for increased testing of such products as well as the establishment of universal guidelines that would facilitate proper regional and crop testing.

### 40.3 Emerging Trends: Biosensors and Precision Agriculture

There are emerging approaches that suggest directions to overcome these limitations, some of which are biosensor and precision agriculture. Such biosensors can measure the density of *B. cinerea* spore at 10^3^ microbial colony‐forming units per gram, which in turn lowers fungicide treatments by about 30% and increases Se‐ChNP effectiveness by 20% in grapes (Li et al. 2021). Optimal humidity of 85%–90% is achieved with the help of humidity sensors in combination with chitosan coatings of strawberries, reducing Rhizopus stolonifer rot by 80% based on real‐time control of environmental conditions (Youssef et al. 2019). Such technologies improve the value of the resources used in that tests have revealed coherent evidence of the reduction of the coating material by 25% and 75% of the pathogen (Sivakumar et al. 2016). Such innovations promise to bridge scalability gaps by optimizing application precision.

### 40.4 Emerging Trends: Genetic Engineering for Resistance

Genetic engineering represents another frontier, enhancing inherent fruit resistance to pathogens. Transgenic apples overexpressing PR‐1 genes reduce *P. expansum* decay by 70%, extending shelf life by 15 days at 20°C (Droby et al. 2009). Grapes engineered for higher SOD activity (up by 50%) show a 60% decrease in *B. cinerea* incidence, complementing *Se‐ChNP* effects (Zhang et al. 2021). While regulatory acceptance lags—only 10% of engineered crops are approved globally—these approaches could reduce reliance on external treatments, cutting costs by 30%–40% (FAO 2017). This trend aligns with sustainable goals but requires public and legal support to realize its full potential.

### 40.5 Recommendations: Standardized Protocols and Broader Applications

To address these challenges, standardized testing protocols are essential. Current efficacy data vary widely—e.g., *Se‐ChNPs* achieve 90% control at 0°C but 50% at 25°C—due to inconsistent methodologies (Pitt and Hocking 2009). Uniform standards, such as ASTM protocols for antifungal testing, could normalize results, ensuring *Meyerozyma guilliermondii* in alginate coatings consistently delivers 80% *P. italicum* reduction across labs (Wang, Li, et al. 2021). Expanding applications to diverse crops is equally critical; *Se‐ChNPs* tailored for grapes need adaptation for citrus, where peel thickness reduces uptake by 40% (Zhang, Zhao, et al. 2020). Trials across 10 fruit types could broaden their efficacy range from 85% to 92% (Sun et al. 2021).

### 40.6 Future Outlook: Balancing Innovation and Feasibility

The future of postharvest disease management hinges on balancing innovation with practical feasibility. A combination of biosensors and genetic engineering could reduce the losses by at least 20%–30% and solve the problem costing approximately $10 billion a year (FAO 2017). However, the cost has to decrease and range from $5 to $1/kg for Se‐ChNPs, and the regulatory bodies must avow the levels of residue (Salem et al. 2022). Incorporation of precision tools into collaborative efforts may remedy this situation as it can involve affordable addresses such as $2/kg of the biological agent (Gotor‐Vila et al. 2017) to reach the spoilage rate at the global level that ranges from 20% to 40% (Gotor‐Vila et al. 2017). These directions present the ways toward achieving sustainable, effective disease control depending on the elimination of the existing economic and technical challenges.

## 41 Conclusion

Nanotechnology and biological control coupled with edible coatings and PGRs provide a suitable approach toward the postharvest diseases of fruits and vegetables, especially those attributable to *B. cinerea* and *P. digitatum* infections. Two examples with such a combination are the Se‐ChNPs with cold storage causing up to 90%–95% reduction in grape decay through an increase of 68% SOD and the destruction of fungal cell walls and biocontrol agents like *Bacillus amyloliquefaciens* incorporated in chitosan coatings, with souring up to 85% of *P. digitatum* through VOCs and nutrient antagonism. Some of the potential control methods of postharvest diseases include edible coatings with added materials like thyme oil, reducing Colletotrichum gloeosporioides rot in mangoes by 92%, while plant growth regulators, including salicylic acid (SA), increase phenolic compounds by 28% in Goji berry, thus reducing decay by 70%. These interventions have, therefore, been designed to improve the quality of storage by eliminating the efficiency of the pathogens and increasing the immune capacity of the host more than each of them doing by 20%–40%. But their popularity is also dependent on high costs ($5–$10/kg for nanotechnology), variation (50% degradation at 25°C), and legal issues; it is a future of cost‐efficient formulations and precision agriculture for fruit supply chains.

## Author Contributions

**Qudrat Ullah:** writing – original draft (equal). **Muhammad Waqar:** conceptualization (equal). **Nimra Sajjad:** formal analysis (equal). **Muhammad Tayyab Arshad:** writing – review and editing (equal). **Sammra Maqsood:** investigation (equal). **Kübra Sağlam:** data curation (equal). **Nimra Maqsood:** formal analysis (equal). **Ali Ikram:** visualization (equal). **Thanet Khomphet:** methodology (equal). **Kodjo Théodore Gnedeka:** validation (equal). **Farhang H. Awlqadr:** writing – review and editing (equal). **Md. Sakhawot Hossain:** writing – review and editing (equal).

## Ethics Statement

The authors have nothing to report.

## Consent

The authors have nothing to report.

## Conflicts of Interest

The authors declare no conflicts of interest.

## Data Availability

The data that support the findings of this study are available from the corresponding author upon reasonable request.
